# Sustainable Production of Biofuels and Biochemicals via Electro-Fermentation Technology

**DOI:** 10.3390/molecules29040834

**Published:** 2024-02-13

**Authors:** María José Salar-García, Víctor Manuel Ortiz-Martínez, Sergio Sánchez-Segado, Raúl Valero Sánchez, Antonia Sáez López, Luis Javier Lozano Blanco, Carlos Godínez-Seoane

**Affiliations:** 1 Department of Chemical and Environmental Engineering, Technical University of Cartagena (UPCT), Campus Alfonso XIII, Aulario C, 30203 Cartagena, Spain; raul.valero@edu.upct.es; 2 Department of Chemical and Environmental Engineering, Technical University of Cartagena (UPCT), Campus Muralla del Mar, 30202 Cartagena, Spain; sergio.segado@upct.es (S.S.-S.); antonia.saez@upct.es (A.S.L.); luisja.lozano@upct.es (L.J.L.B.); carlos.godinez@upct.es (C.G.-S.)

**Keywords:** electro-fermentation, microbial metabolism, bioelectrochemistry, biomass, added-value chemicals, biofuels

## Abstract

The energy crisis and climate change are two of the most concerning issues for human beings nowadays. For that reason, the scientific community is focused on the search for alternative biofuels to conventional fossil fuels as well as the development of sustainable processes to develop a circular economy. Bioelectrochemical processes have been demonstrated to be useful for producing bioenergy and value-added products from several types of waste. Electro-fermentation has gained great attention in the last few years due to its potential contribution to biofuel and biochemical production, e.g., hydrogen, methane, biopolymers, etc. Conventional fermentation processes pose several limitations in terms of their practical and economic feasibility. The introduction of two electrodes in a bioreactor allows the regulation of redox instabilities that occur in conventional fermentation, boosting the overall process towards a high biomass yield and enhanced product formation. In this regard, key parameters such as the type of culture, the nature of the electrodes as well as the operating conditions are crucial in order to maximize the production of biofuels and biochemicals via electro-fermentation technology. This article comprises a critical overview of the benefits and limitations of this emerging bio-electrochemical technology and its contribution to the circular economy.

## 1. Electro-Fermentation as a Sustainable Platform for a Circular Bioeconomy

The promotion of renewable resources is a must to move from an economy based on the use of fossil fuels to a sustainable and circular economy. The European Bioeconomy Strategy for the period of 2018–2030 [1] highlights these challenges and declares the necessity for a change into a resource-efficient production scenario with a simultaneous valorization of and reduction in waste streams. In the last years, many efforts have been devoted to developing new technologies to exploit renewable resources in order to accomplish this transition. Biofuels such as biodiesel, biomethanol, bioethanol or biogas, and energy vectors such as hydrogen play an important role in the decarbonization process of society. Biofuels can be used as fuel additives or in their pure form whereas hydrogen can be electrochemically transformed into electricity via fuel cell technology. All these alternatives to fossil fuel might help to reduce greenhouse emissions and global warming as well as contribute to achieving sustainable development goals [2]. In this context, biotechnology plays a key role in the production of biofuels and value-added chemicals using raw materials such as biomass through the pretreatment, conversion, separation, and purification processes [3]. Many of these consist of the transformation of monosaccharides or disaccharides such as sucrose through various chemical and biochemical routes including dehydrogenation–hydrogenation, reforming, and microbial and intracellular fermentation [4].

Specifically, fermentation has become a prominent technology not only in the food industry but also in other sectors to produce a wide variety of commodities such as vitamins, enzymes, probiotics, solvents, biofuels (e.g., ethanol), biopolymers (e.g., polyhydroxyalkanoates -PHA- like polyhydroxybutyrate- PHB), and acids (e.g., lactic acid), among others. Industrial fermentations are nevertheless affected by time-consuming and capital-intensive operations related to upstream stages such as the conditioning and purification of substrates and the need for maintaining sterile conditions [5]. Conventional fermentation, on the other hand, is greatly controlled by feedback inhibition which represents a major constraint. The accumulation of generated products such as organic acids can modify the physiological capacities of microbial cells, altering their metabolism and growth [6]. Innovative strategies have been researched to approach these drawbacks including the combination of conventional anaerobic digestion with photofermentation [7] or the use of bioelectrochemical systems (BESs) to control stability and optimize chemical production.

The fact that microorganisms can be electrically connected to devices has been known since 1911 when Potter first proved that an electrical current could be generated by certain types of bacteria by converting the chemical energy present in organic substrates into electricity [8]. However, it has been only over the last two decades that microbial electrochemistry has become a revolutionary new technological approach through intense research. BES agglutinates a set of systems in which microorganisms and other biocatalysts are integrated with an electrochemical technique to boost and control the oxidizing and/or reducing metabolism pathways. Microbial fuel cells (MFCs) [9] for power generation and microbial electrolysis cells (MECs) [10] for the production of value-added products like H_2_ or H_2_O_2_ are well-known classes of BES systems. In both cases, power generation or chemical production can occur with simultaneous water treatment.

**Figure 1:**
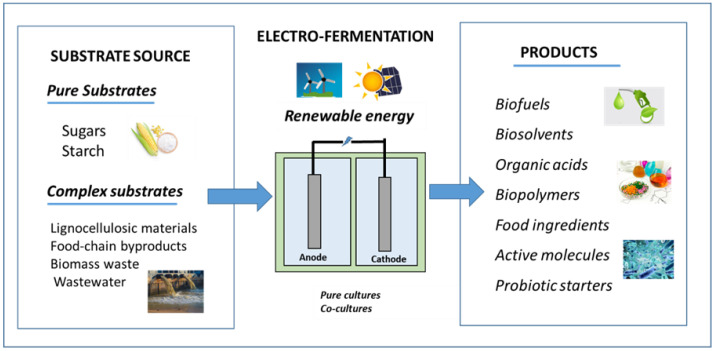
Electro-fermentation as a sustainable platform for chemical production and waste valorization.

In the last decade, electro-fermentation (EF) has drawn the attention of the scientific community as a potential alternative to conventional anaerobic digestion processes [6,11,12]. As in the rest of BESs, electrodes are key components, in this case, for the optimization of microbial fermentative routes. Electrodes can work as both electron sources and sinks modifying the medium by altering the redox balance [13]. The concept of EF was well established in recent years by Rabaey et al. [14,15]. This technique can be exploited to offer an enhanced control of the process to obtain products of a higher purity in comparison to conventional fermentation, improving the microbial cell growth stability and density. It can be also used to attain chain elongation, e.g., the production of medium-chain fatty acids from volatile fatty acids. As in the conventional process, EF consists of the fermentation of energy-rich substrates like alcohols and carbohydrates, but under the application of an external potential that triggers oxidative/reductive pathways [5] (see Figure 1).

The possibility of working under unbalanced conditions is greatly advantageous for the improvement of fermentation yields. In this respect, thermodynamics represents a limitation in fermentative processes since many of the overall reactions taking place in fermentation can be considered spontaneous. The major constraint to obtaining high yields lies in cell regulation, which keeps the metabolism in redox balance. The introduction of electrodes into the fermentation media will ultimately cause the shift from balanced conditions to unbalanced operation, allowing for, in theory, the stoichiometric conversion of the substrate into the final product. Therefore, EF technology can offer higher yields than the theoretical ones obtained from fermentation under balanced conditions [16]. Table 1 summarizes the most relevant aspect of the fermentation and electro-fermentation processes as well as their potential practical applications.

A typical EF system is formed by an anodic chamber and a cathodic chamber electrically connected through an external circuit. This electrochemical configuration can be operated in two modes, (i) anodic EF and (ii) cathodic EF. The anodic EF mode refers to a system in which the final product is more oxidized than the substrate. This applies, for example, to the case of the obtention of ethanol using glycerol as a substrate. The working electrode of the cell is the anode, which allows for the dissipation of electron excess (electron sink). On its part, cathodic EF implies the formation of a reduced final product. The working electrode is now the cathode, which provides electrons (electron source). The generation of butanol from glucose is an example of cathodic EF. One of the differences between EF systems and other bioelectrochemical systems such as MECs is that the amount of current density to be provided to the system is lower to cause a significant effect on the process [13].

EF requires the use of electroactive bacteria, that is, microorganisms that can interact with extracellular environments or elements to exchange electrons (extracellular electron transfer). *Geobacter sulfurreducens* and *Shewanella oneidensis* are recognized electroactive bacteria exploited in BES systems due to their capability to perform direct electron exchanges with electrodes [17]. These microorganisms colonize and grow over electrode surfaces forming an electroactive biofilm. The main cell components responsible for extracellular electron transfer are exposed multi-heme cytochromes. Nanowires and other redox proteins can also intervene in these transfer mechanisms [18]. The use of chemical compounds is another option to perform an indirect electron transfer between bacteria and electrodes. In this case, such compounds, known as mediators or electron shuttlers, are redox-active and assist in electron transport. These diffusible redox compounds can comprise self-excreted cell molecules, externally added artificial mediators, and primary metabolites [19].

The successful production of several chemicals and biofuels such as short-chain and medium-chain fatty acids, hydrogen, biogas, bioalcohols, and organic acids have been demonstrated via EF technology [5,20,21]. However, although this technology offers great potential, EF is still at an early development stage and requires a great deal of research effort for its practical implementation and widespread use in the industry. The optimization of the electron transfer between the electroactive bacteria and the electrodes in EF systems is of crucial relevance for the advancement, upscaling, and commercialization of this technology. Research strategies also focus on the study of substrates, reactor configurations and materials, microbial catalyzers, and targeted products. Thus, this article offers a review of the last advances in the field, beginning with the basic principles down to the current advantages and limitations provided by EF.

## 2. Principles of Electro-Fermentation

Fermentation is the result of a series of oxidation and reduction reactions that are maintained in balance. A key parameter in the process is the oxidoreduction potential or ORP. The ORP expresses the oxidation and reduction capacity of the fermentation medium, but it is also a measurement of the metabolic activity of bacteria [22]. The extracellular and intracellular ORPs are different because of the cytomembrane separation and cellular redox homeostasis. Extracellular ORP is affected by the fermentation medium composition and other parameters such as temperature. This is of key importance since it directly influences the intracellular potential through changes in the ratio of nicotinamide adenine dinucleotide (NAD) in reduced and oxidized forms (NADH and NAD+, respectively). Since the control of the redox homeostasis at the cell level is vital for metabolism functioning, a change in the extracellular potential leads to the adjustment of the electron flow in the metabolism by changing the NADH: NAD+ ratio [18,23]. The modification of the metabolic pathways can be then controlled by different strategies in order to shift these pathways to the generation of a desired product. These include the control of extracellular ORP by enzyme synthesis and genetic engineering [24] and chemical methods [25]. Thus, EF is an alternative method to regulate extracellular potential and metabolic pathways through the integration of electrodes in the fermentation media for the collection or supply of electrons.

**Figure 2:**
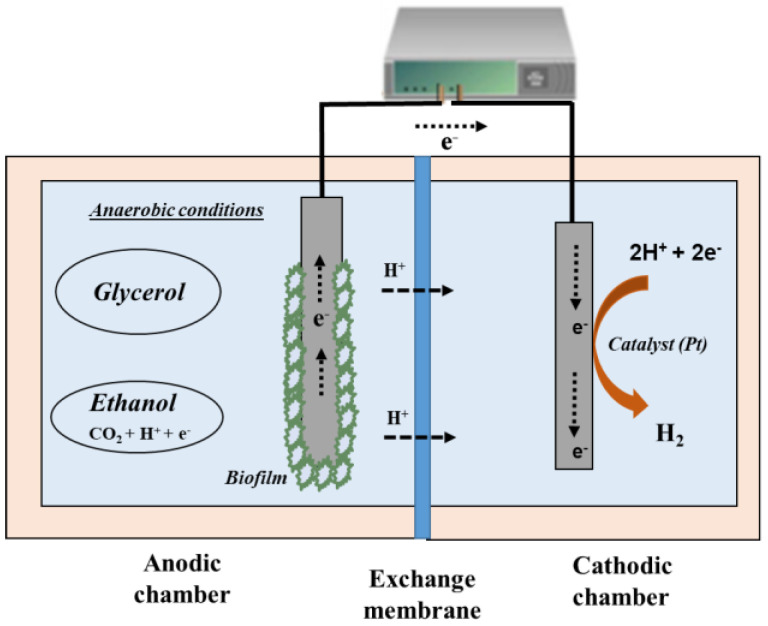
Example of the anodic electro-fermentation of glycerol to obtain ethanol.

Basically, in the anodic EF, a substrate is oxidized into a certain product and a portion of the electron excess is released in the anode chamber via the extracellular electron transfer mechanism (see Figure 2). The electrons released at the anode cause the reduction of the above-mentioned ratio NADH: NAD+, which leads to pathways that regulate NADH at the cellular level. Thus, when electroactive microorganisms are present in the fermentation medium, they can directly transfer the generated electrons to the electrode material via interspecies transfer mechanisms [5]. Through the maximization of the subtraction of electrons via the oxidation of the intermediate metabolic electron acceptors, the consumption of NADH is promoted, and thus the creation of proton gradients for the generation of adenosine triphosphate (ATP), the energy-carrying molecules in the cells [26].

**Figure 3:**
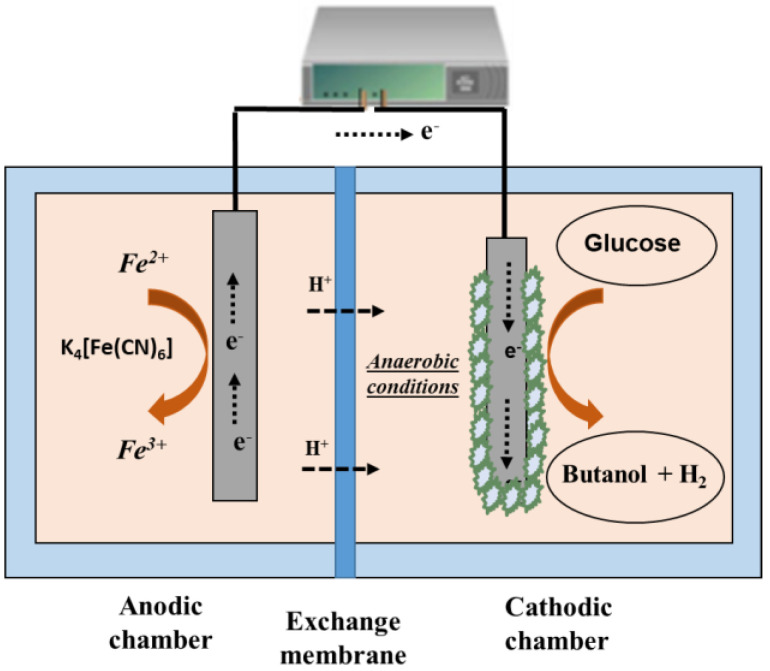
Example of the cathodic electro-fermentation of glucose to obtain butanol using a redox mediator in the anode chamber [28].

In the cathodic EF, electrochemically assisted fermentation is performed in the cathode chamber (see Figure 3). In such cases, microorganisms utilize the cathodic electrode as an electron source or electron donor, and substrates act as acceptors of electrons. The presence of additional reductants in the fermentation medium can also contribute to the shift of the conventional routes [27].

According to Schievano et al. [29], the fermentation pathways in EF can be electrochemically improved through the boosting of certain electron transport routes and the improvement of energy conservation mechanisms like ATP formation. On the other hand, both the addition of inorganic and organic molecules in the fermentation media can act as mediators to boost electron transfers and serve as final or intermediate acceptors. For example, soluble redox mediators such as methyl viologen, humic acids, thionin, or riboflavins can act as mediators to shuttle electrons from electrodes and vice versa. These mediators can be used when microorganisms are not fully electrochemically active.

EF can be performed with pure microorganism cultures or with mixed cultures. *Geobacteraceae* and *Shewanellaceae*, as well-known electroactive bacteria, can be exploited in these systems since they can directly interact with the anodic and cathodic electrodes for electron transfer via the formation of biofilms over electrode surfaces [30]. The specificity of microorganisms towards certain substrates is another key factor that must be taken into consideration. For example, *Clostridium perfringens* is a gram-positive, spore-forming, and rod-shaped bacterium that is recognized as electroactive, and at the same time, it is capable of consuming a broad variety of energy-rich substrates such as carbohydrates and alcohols [31]. However, both features can be found only in a few cases, and thus, mixed cultures can be needed in EF systems. Through the use of electroactive and fermentative co-cultures, both the targets, electron transfer, and the substrate conversion into the desired products can be achieved. The work of Moscoviz et al. [32] can be cited as a representative case of this strategy. In this study, the authors demonstrated the cooperative growth of co-cultures for glycerol fermentation. The use of mixed cultures is also interesting for other synergetic effects. The production of growth factors, the removal of certain inhibitors, and the creation of specific environmental conditions by one culture may be advantageous for others. Moreover, multiple synthesis pathways in cascade to produce a certain metabolite might not be viable by one type of microorganism, while their combined effects can be approached to overcome this limitation [29].

On the other hand, the electrode materials can greatly affect the efficiency of EF, as will be discussed later in detail. In summary, electrodes need to be optimized in order to provide an efficient structure for biofilm development and electron exchange. Ideally, electrodes must offer a high conductivity, biocompatibility, a lack of corrosiveness, and a high specific surface and porosity. On the other hand, catalysts over electrodes including noble and non-noble metals can be used to reduce the overpotential of redox reactions [20].

Finally, it is interesting to have indicators to express the overall efficiency of an EF process. For this case, we can follow the definition provided by Moscoviz et al. [13] to differentiate between general BES and EF processes. As remarked by these authors, there are cases in which a substrate like glucose can be used for both metabolite production (hydrogen) and electricity in BES. In such cases, electrodes can be exploited for the optimization of electricity generation. Thus, Moscoviz et al. [13] defined EF efficiency according to Equation (1) to assess the energetic performance of an EF process, in which the production of a chemical or metabolite is the final objective.

(1) $${EF}_{efficiency}=\frac{Q_{e}}{Q_{product}}$$

where *Qe* is the result of the integral of the electric current (*I*) applied during EF operation, that is, the charge transfer across the electrical circuit, and *Q_product_* is the total charge amount in the product. *Qe* can be calculated according to Equation (2):

(2) $$Q_{e}=\int_{0}^{t}I\times dt$$

where *I* is the electrical current (A) and t refers to the time (s).

On its part, *Q_product_* can be calculated according to Equation (3):

(3) $$Q_{e}=N_{product}{\;\times \;n}_{product\;}\times \;F$$

where *N_product_* is the number of electron moles available per mole of product which can be calculated by multiplying the number of atoms of C, N, O, and H (w, x, y, and z, respectively) in the organic molecule structure by the oxidation state of each element as follows:

N(C_w_N_x_O_y_H_z_) = 4w − 3x − 2y + z

*n_product_* is the number of target product moles, and *F* is the Faraday constant (96,485 C·mole^−1^).

If EF efficiency ranges between 0 and 1, a higher number of electrons are recovered in the target product than those provided or consumed to/from the external current supplier. By contrast, if EF efficiency shows values higher than 1, it means that either an anodic EF is operating as an MFC, thus producing electricity, or that a cathodic EF is working as an MEC, thus consuming electricity.

## 3. Process Design and Electro-Fermenter Configurations

Like other bioelectrochemical technologies, the design of the electro-fermenter including the materials and operating conditions is directly related to the overall cost of the system as well as the efficiency of the process. Different configurations of anaerobic fermenters using an external power supply are used to perform the electro-fermentation process with the electrode material along with the reactor configuration being two of the most important factors to improve the performance of the device. Electro-fermenters can be grouped into two main categories depending on the place where the reactions occur: (i) single-chamber reactors, where the anodic and cathodic reactions are carried on in the same chamber, and (ii) double-chamber reactors, where the reactions take place in membrane-separated chambers. Despite double-chamber electro-fermenters allowing the electrode reactions to occur independently, the internal resistance caused by the distance between the electrodes and presence of the ion exchange membrane, whose internal resistance ranges between 1 and 10 Ω.cm^−2^, is higher than in a single-chamber reactor, which increases the voltage losses, reaching values between +0.26 and +0.38 V. In addition, the transference of other cations apart from protons through the proton exchange membrane causes a pH gradient, which also results in a voltage loss of around 0.06 V per pH unit [6]. By contrast, single-chamber reactors are a simpler alternative than double-chamber systems. Additionally, this configuration offers a lower internal resistance due to the proximity between the electrodes, which implies lower voltage losses. However, the metabolites produced require a post-treatment process. Regardless of the reactor configuration, both pure (e.g., glucose, lactate, acetate, etc.) and complex compounds (e.g., synthetic waste, waste-activated sludge, food waste) have been used as substrates to simultaneously produce biogas (H_2_ and/or CH_4_) and organic acids via electro-fermentation, being the simplest fuels and the most commonly used so far. Electrodes play an essential role in electro-fermentation since they act as an electron donor or acceptor, depending on the operating mode, which contributes to regulate the overall microbial metabolism. The anode can act as an electron mediator, which will help to optimize electro-fermentation performance [33]. Similar to other BES technologies, carbon-based materials are the most widely used to prepare the electrodes due to their porosity, high surface area and conductivity, which favors redox reactions, good biocompatibility, which promotes biofilm growth, and their lower cost compared to metal-based electrodes [34]. The following sections comprise the most common reactor designs used to bioelectrochemically produce different types of biogases and/or organic acids which include the reactor configuration, electrode material, type of membrane, nature of the substrate and inoculum as well as the type of polarization of the electrodes.

### 3.1. Single-Chamber Electro-Fermenters

As previously commented, membrane-less single-chamber electro-fermenters have been reported in the literature to be a suitable configuration to transform different kinds of substrates into methane, hydrogen, or bioethanol, among others. Table 2 summarizes some of the recent works reported regarding electro-fermentation processes performed in single-chamber reactors In 2015, Zhao et al. [35] applied an external voltage of 0.6 V between a graphite-rod cathode and a graphite-brush anode, both immersed in a cylindrical reactor inoculated with activated sludge for producing methane. After 33 days, the accumulative methane production of the electro-fermenter was 2998.4 mL, whereas the conventional fermentation reached only 904.5 mL. Their results demonstrate the improvement in methane production achieved by using polarized electrodes during the fermentation process. Then, in 2016, Zhen et al. [36] also used a single-chamber reactor with the cathode submerged to evaluate the effect of applying different voltages on methane production as well as the *E. densa* fermentation process. The maximum amount of methane was produced at 1.0 V (248.2 ± 21.0 mL.L^−1^.d^−1^). Their results showed that the syntrophic and beneficial interaction between fermenting bacteria and electroactive bacteria results in process stabilization and improves energy recovery. Later on, Ren et al. [37] reported the positive effects of adding graphite particles in an electro-fermentation methanogenesis process. The electro-fermenter set-up consists of a three-electrode system made of graphite and working at a constant potential of −0.6 V (vs. SCE) and fueled with sodium acetate. The authors obtained an improvement in the characteristics of the cathode biofilm attributed to the enrichment of the reactor with 0.1 g of graphite which also resulted in an increase of 54.3% in methane production (from 1.99 to 3.07 mmol) after 18 days of operating. The presence of the graphite particles not only increased the number of active sites and redox groups on the biofilm, which improved the electron transport process, but also boosted the development of methanogens and phylum proteobacteria, enhancing the electro-fermentation methanogenesis process.

In addition to the production of different types of biogases, single-chamber electro-fermenters have also been used to simultaneously produce fatty acids such as acetic acid, lactic acid, or butyric acid, among others. In particular, Shanthi Sravan et al. [38] constructed an air-cathode single-chamber electro-fermenter fueled with food waste with an organic loading of 10 g.L^−1^. Applying a set potential of −0.6 V (vs. Ag/AgCl), the production of volatile fatty acids was higher (4595 mg.L^−1^) compared to the production when the system operates in a closed circuit under an external resistance of 100 Ω (3593 mg.L^−1^). Among the different fatty acids produced, acetic acid prevailed over butyric acid and propionic acid. Regarding the biogas generation, it was also favored by applying the set potential and showed the following trend: H_2_ (26%) > CH_4_ (4%). Finally, the electro-fermenter design also allowed the production of biohythane when the biogas generated contained a specific ratio of H_2_/CH_4_. More recently, in 2021, d’Ippolito et al. [39] used a single-chamber three-electrode reactor to perform the glucose electro-fermentation of *T. neapolitana* in hyperthermophile conditions. Their results showed that an electroactive biofilm grew on the carbon-based electrodes at 80 °C which reacted to a dynamic voltage applied. Among the different conditions studied, a cyclic potentiodynamic polarization of ±0.8 V (vs. Ag/AgCl) favored the biofilm growth and bacteria attachment coupled with a production of lactic acid of 33 mM. By contrast, a higher dynamic voltage (±1.2 V) caused bacteria detachment and resulted in a lower amount of lactic acid being produced (12 mM). Regarding the acetate and hydrogen production, the yield of both products was higher under the potentiodynamic conditions than that achieved with the potentiostatic polarization. A similar electro-fermenter setup was used by Paiano et al. [40] to obtain butyric acid. In this case, a membrane-less reactor was inoculated with anaerobic sludge and fed with a mixture of glucose, acetate, and ethanol. Two graphite rods were used as electrodes and a range of voltage from 0.6 V to 1.5 V was applied between them. Among the different conditions studied, the highest production of butyric acid was obtained at an applied voltage of 1.3 V, reaching an increase of 2.7-fold compared to the value obtained in the open circuit potential. When the voltage applied was larger than 1.4 V, a reduction in the butyric acid produced coupled with an increase in energy consumption was observed. The results obtained are in line with those observed in a double-chamber reactor working with a membrane as a separator under potentiostatic polarization. Furthermore, the nature of the inoculum might not affect the performance of the electro-fermentation.

### 3.2. Double-Chamber Electro-Fermenters

Some alternatives to single-chamber reactors are double-chamber systems including a proton exchange membrane as a separator (see Table 3). Among the early works concerning this type of reactor configuration was that performed by Sasaky et al. [42] in 2013. The authors evaluated the effect of the cathodic reaction on the production of methane and volatile fatty acids as well as on the chemical oxygen demand removal over the results obtained in a non-bioelectrochemical reactor and a stirred tank reactor. In all cases, the same reactor set-up was used, which consisted of a cylindrical glass vessel comprising two concentric chambers separated by a Nafion^®^ 117 (Dupont, DE, USA) membrane. The outer chamber was used as a cathodic compartment where eight carbon plates were used as working electrodes. The main difference between the three methanogenic reactors studied was that the electrode side facing the counter chamber was covered with carbon fiber fabric in the bioelectrochemical and non-bioelectrochemical systems but not in the stirred tank reactor. A carbon bar was used as a counter electrode and was submersed in 170 mL of 100 mM NaCl being the volume of the cathodic chamber and 13.3-fold the volume of the anodic chamber. All the systems were fed with thickened sewage sludge and the results showed that the bioelectrochemical system reached a maximum gas production rate of 3.57 L.L^−1^.day^−1^ at a hydraulic retention time (HRT) of 4.0 days, which is more than 3.5 times higher than the production rate of the non-bioelectrochemical reactor at the same HTR, which demonstrates the positive effect of the potential applied (−0.8 V vs. Ag/AgCl) on the gas production. The lowest methane production was observed in the stirred tank reactor (1.37 L.L^−1^.day^−1^ at an HRT of 12 days) coupled with a degradation of the system which also suggests the benefits of using carbon fiber fabric. On the contrary, a pure culture of *C. pasteurianum* fed with standard minimum media enriched with glucose was used to produce biobutanol in an electro-fermenter by Khosravanipour Mostafazadeh et al. [43]. The set-up consisted of an H-type reactor equipped with graphite felt as electrodes and Nafion^®^ 117 as a selective separator. The results were also compared with those obtained using stainless steel electrodes. Among the different voltages applied, the results showed that an applied voltage higher than 1.5 V results in a reduction in the butanol produced for both types of electrode materials. These results might be due to the increase in hydrogen production as the voltage also increased due to the competition between both pathways and the instability of the biofilm. Regarding the electrode material, the production of butanol was higher using the graphite felt instead of the stainless steel as electrodes for all the voltages applied, which also confirms the benefits of using carbon-based materials, as previously commented. According to the central composite design, the maximum amount of butanol produced would be 13.31 g.L^−1^ under a set voltage of 1.32 V. Later, Villano et al. [44] also used an H-type reactor equipped with the same commercial proton exchange membrane, two graphite rods as electrodes, and an Ag/AgCl reference electrode (+199 mV vs. SHE) to analyze the bioelectrochemical production of isobutyrate. The reactors were inoculated with anaerobic sludge and fed with different carbon sources individually supplied or combined (glucose, ethanol, and acetate) and a set voltage of −0.7 V (vs. SHE) was applied to polarize the electrodes. According to the carbon source, the yield of isobutyrate produced (mol/mol glucose) under the closed circuit showed the following trend: glucose + acetate > glucose + acetate + ethanol > glucose > glucose + ethanol. In all cases, the applied voltage boosted the isobutyrate production but also the combination of glucose and ethanol. It is worth noting that the presence of acetate in both the binary or ternary mixtures results in an increase in the isobutyrate production from the glucose and a reduction in the yield of hydrogen generated. Furthermore, the results showed that the yield of isobutyrate produced significantly increased after polarizing the electrodes, reaching a maximum value of 0.43 mol/mol glucose, which is 20-fold higher than that obtained under open circuit conditions when acetate is a co-substrate. This work reported for the first time the synergetic effect of applying a set voltage and the presence of acetate as a co-substrate on the production of isobutyrate via glucose fermentation. A similar configuration including a double-chamber reactor and a Nafion^®^ 117 membrane was used to produce propionate via lactate electro-fermentation by Isipato M. et al. [45]. In this case, the cathode was made of carbon cloth whereas the anode consisted of a platinized titanium mesh. The system also included an Ag/AgCl reference electrode, and it was inoculated with anaerobic sludge from a dairy processing plant, whereas the D-lactate, butyrate, or both were added to the catholyte. Their results show that an applied voltage of −1 V (vs. Ag/AgCl) boosted the propionate production at a pH of 5 and an initial concentration of lactate of 20 mM reaching up to 0.44 g.L^−1^.d^−1^, whereas the maximum propionate production rate (0.96 g.L^−1^.d^−1^) was obtained from an initial lactate concentration of 150 mM with less than 1 kWh.kg^−1^ of propionate of energy consumed. These results might be related to the presence of *Tyzzerella* sp. And *Propionibacterium* sp., well known as propionate-producing microorganisms. In addition, the presence of other microorganisms, such as *Desulfovivrio* sp. And *Acetobacterium* sp., in the cathode was involved in converting the CO_2_ generated in the process into acetate which not only increases the yield of the volatile fatty acids but also reduces the carbon emissions contributing to the development of sustainable chemical processes.

Cation exchange membranes such as CMI7000 (Membranes International Inc., Ringwood, NJ, USA) have also been used as membranes in electro-fermenters as an alternative to Nafion^®^. For instance, Jiang et al. [46] used this type of separator in a double-chamber reactor where graphite felt was used to elaborate both the working and the counter electrode along with an Ag/AgCl reference electrode (+0.2 V vs. SHE). The system was inoculated with anaerobic sludge and fed with glucose-rich synthetic wastewater. This work reported that under neutral pH conditions, the more negative the working potential the higher the production of methane, hydrogen, and acetic acid, whereas an increase in the set potential results in a significant reduction in the methane and acetic acid production. However, the effects of changing the applied potential on the distribution of the products are buffered by a slight acidification of the media (pH: 6.2). Under neutral conditions, the amount of methane produced ranged from 4.3 to 6.7 mL at a set potential of −1.0 V, but the production increased as the pH decreased by 6.2. These results demonstrate that the metabolite distribution might be fine-tuned by varying the applied potential and the pH of the media which favors the control of the whole process.

### 3.3. Hybrid Configurations of Electro-Fermenters

As previously commented, MFCs are BESs that convert the chemical energy stored in substrates of different natures into electricity using the microbial metabolism. This technology might provide the energy required to perform the electro-fermentation process, which allowing researchers to design a self-sufficient system to transform carbon-rich substrates into different value-added products such as hydrogen, methane, or volatile fatty acids, among others. In 2015, Chandrasekhar et al. [47] designed a self-driven membrane-less air-cathode single-chamber electro-fermenter to transform solid food waste into bioelectricity, biohydrogen, and bioethanol. The system was able to reach a maximum power output of 162.4 mW.m^−2^ on day 9, 21.9 mL.h^−1^ of hydrogen on day 19, and 4.85% *w*/*v* of bioethanol on day 20 of operation. Their results confirmed that the production of hydrogen might be due to a mixed acetate/butyrate-type fermentation, whereas the presence of other metabolites such as formate or lactate could drive other metabolic pathways which reduce the hydrogen production. Simultaneously, Nikhil et al. [48] combined MFC technology with acidogenic fermentation in a novel single-chamber biocatalized electro-fermenter. The hybrid system comprised two pairs of graphite electrodes in a single-chamber reactor. Whereas the anodes were submerged, the cathodes were air-faced. This novel prototype of MFC–EF allowed them to reach a maximum power output of 72 mW.m^−2^ and produce 343 mL of biohydrogen using synthetic wastewater as a substrate with an organic load of 5000 mg.L^−1^ when operating in the close circuit under an external resistance of 300 Ω.

Some alternatives to avoid the power supply are submersible MECs which combine MEC technology with anaerobic fermentation. This configuration consists of coupling two cathodic chambers to an existing anaerobic digester. Where one of the cathodic compartments is mainly used to produce electricity, the other is used to produce hydrogen. In 2013, Van Eerten-Jansen et al. [49] were able to reach a hydrogen production rate ranging between 5 and 487 mL.d^−1^ using platinum-coated titanium mesh anodes and graphite felt cathodes, both separated by a cation exchange membrane.

From another point of view, electro-fermentation might be used as a hydrogen supplier for pyrolysis. So far, the hydrogen required in the last step of pyrolysis of biomass usually derives from fossil fuel which releases greenhouse gases into the atmosphere and contributes to global warming. Electro-fermentation brings the opportunity to transform the pyrolysis process into a more sustainable process [5,20]. However, the benefits might be used the other way around since electro-fermentation might also be used to revalorize the aqueous waste stream obtained during the pyrolysis process named the bio-oil aqueous phase (BOAP). In this case, electro-fermentation might be a suitable option to transform BOAP into hydrogen or value-added products [50].

## 4. Production of Platform Chemicals through Electro-Fermentation

As commented above, EF technology uses electricity for the synthesis of chemicals with the aid of microorganisms attached to the cathode/anode surface which act as biocatalysts able to perform oxidation-reduction reactions as effectively as other chemical catalysts. The biocatalyst, reduction potential, electrochemically redox mediators, and the type of bioelectrode play an important role in the synthesis of biochemicals and biofuels [14,51]. The following sections comprise the production of different organic acids as well as alcohols during EF.

### 4.1. Organic Acids

During EF, pure or mixed microbial cultures convert organic and inorganic substrates including CO_2_ in short-chain (C2–C5) organic acids through the acidogenesis process either in the cathode or the anode, as previously commented [52]. During biochemical production, acetate is regarded as the main intermediatory molecule [53]. The first discovery of electroacetogenesis was demonstrated by Nevin et al. [54,55]. In their works, they reported the possibility of reducing CO_2_ into acetate through electro-fermentation systems testing different acetogenic bacteria at potentials of −0.4 V vs. SHE and −0.6 V vs. SHE. Gyldemyn et al. [56] studied the effect of different ion exchange membranes on the production of acetate from CO_2_ at a constant potential of +0.2 V vs. SHE using a mixed microbial culture dominated by *Clostridiales*. They demonstrated that systems with anionic exchange membranes allowed for the in situ recovery of acetate leading to a 32% higher production rate and recovery than those systems that did not account for product recovery. Moreover, Kracke et al. [57], developed a novel transition metal cathode to produce H_2_ to be used as an electron mediator to enhance the CO_2_ conversion to acetate by *Sporomusa ovate* at −0.6 V vs. SHE.

Other substrates have also been tested for the production of acetate. Sturm-Richter et al. [58] reported a new and accelerated way for anaerobic glycerol fermentation by connecting the central metabolism of *E. coli* to an anode surface. *E. coli* was engineered to enhance the electron transfer to the anode using methylene blue as a suitable electron shuttle improving glycerol conversion into acetate at a rate of 0.3 mmol of acetate per mol of glycerol consumed using a constant potential of +0.2 V vs. SHE. TerAvest et al. [59] investigated the acetate production from lactate using an engineered modified strain of *E. Coli* to allow for the direct transfer of electrons to the electrode. They found that at a constant potential of +0.2 V vs. Ag/AgCl the engineered strain was able to produce two times more acetate than the control test.

Organic acids such as propionic, lactic, and butyric acid have been electricity-driven produced. Schuppert et al. [60] carried out the EF of lactate with the microorganism *Propionibacterium acidi-propionici* using cobalt sepulchrate as an electron donor at −0.47 V vs. SHE. In their work, they found that propionate was the only fermentation product either in a batch or continuous operation. Xu et al. [21] demonstrated the potential of cathodic EF for the enhancement of the productivity and optical activity of lactic acid from activated sludge and food waste using a mixed microbial community at a constant potential of +0.1 V vs. SHE. They concluded that the in situ electron supply through the cathode was successfully increasing the productivity 4.73 times and the optical activity from 3.6 to 42.3% towards L-lactic production. Choi et al. [61] examined the production of butyrate from cultures of *Clostridium tyrobutyricum* BAS 7 from sucrose at −0.4 V vs. Ag/AgCl using neutral red as an electron mediator. The study demonstrated an increase of 1.7 and 1.3 times in the productivity and the yield of butyrate, respectively; meanwhile, Ganigué et al. [62] have proven for the first time the bioelectrochemical production of butyrate from CO_2_ at −0.8 V vs. SHE. They demonstrated that the reduction of CO_2_ to butyrate was hydrogen-driven and that there was the possibility to also produce ethanol and butanol at low pH values.

Other researchers have focused on microorganisms’ modification to induce electroactivity and metabolic pathways toward a target product. For instance, Wu et al. [63] engineered an *E. coli* strain to induce electroactivity and succinate production for the first time. They conducted EF experiments using neutral red as an electron mediator for succinate production from glucose at −0.65 V vs. Ag/AgCl achieving a production yield of 1.10 mol of succinate per mol of glucose. Kim et al. [64] carried the genetic modification of *Klebsiella pneumoniae* L17 to produce 3-hydroxypropionic acid from glycerol at +0.5 vs. Ag/AgCl using 2-hydroxy-1,4-naphthoquinone as an electron mediator. They reported a 3-hydroxypropionic acid production 1.7 times higher than the control without the applied potential.

Medium-chain fatty acids (MCFAs) are hydrophobic, have low solubility, and have an easy separation from fermentation broth. The biological production of MCFAs through the reverse oxidation pathway requires a carbon source such as ethanol, acetate, lactate, or butyrate for electricity-driven chain elongation using an in situ potential [20]. Van Earten-Jessen et al. [49] used mixed cultures to convert waste biomass into MCFAs. They investigated whether the cathode of a BES can be used as the electron donor for the conversion of acetate into MCFAs demonstrating that MCFAs were produced in a BES at −0.9 V vs. SHE cathode potential, without the addition of an external mediator. Caproate, butyrate, and smaller fractions of caprylate were the main products formed from the acetate.

### 4.2. Alcohols

In electro-fermenters, electrons produced in the anodic chamber can be utilized to reduce organic acids to bio-alcohols. The rate of these reactions and the electro-metabolic control of the entire mechanism is strongly dependent on the biocathode [65]. Biofuels such as ethanol and butanol can be produced in electro-fermentative systems. Fynn et al. [66] demonstrated the use of electrode-based electron acceptors to balance redox reactions in biotransformations. For that, they engineered the metal-reducing bacteria *Shewanella oneidensis* to stoichiometrically convert glycerol into ethanol through the removal of two electrons used in the external reduction reaction of the electrode at a fixed potential of +0.44 V vs. SHE.

Speers et al. [66], carried out the anodic EF of glycerol into ethanol using a mixed consortium of the exoelectrogen *Geobacter sulfurreducens* and the bacterium *Clostridium cellobioparum*. *Clostridium cellobioparum* ferments glycerol into ethanol in high yields (90%) along with other fermentation by-products which are used as electron donors for *Geobacter sulfurreducens* to produce hydrogen. They found that this synergistic association increases glycerol consumption by up to 50 g.L^−1^ and ethanol production by up to 10 g.L^−1^, exceeding the bio-anode capacity to remove fermentation by-products leading to the production of 1,3-propanediol that acts as a sink for electron excess. Awate et al. [67] tested the anodic EF of cellobiose using *Cellulomonas uda* to produce ethanol and a genetically modified consortium of different strains of *Geobacter sulfurreducens* to remove non-ethanol fermentation by-products at +0.24 V vs. Ag/AgCl in a single-chamber system. They demonstrated the effectiveness of the process, achieving enhancements the yields and productivity of 2.7 and 3.7, respectively, in comparison to standard fermentation.

Biological acetate reduction with hydrogen is a potential method to convert wet biomass waste into ethanol. Steinbusch et al. [68] investigated the acetate reduction using an electrode instead of hydrogen as an electron donor and methyl viologen as an electron mediator. They found that by using a flat-plate configuration with a fixed potential of −0.55 V vs. SHE and methyl viologen ethanol, production and efficiency were increased 6 and 7.5 times, respectively, compared to the control test. However, hydrogen was co-produced at the cathode making it unclear if the acetate reduction was a consequence of the electrons supplied by the mediator or by the hydrogen.

Bio-based butanol is considered a significant platform chemical because of its interesting properties as a substitute for gasoline. Traditional butanol fermentation has several limiting factors of which the most significant one is its low productivity which could be overcome by novel techniques such as biolectrosynthesis [20]. Choi et al. [69] carried out the cathodic EF of glucose using *Clostridium pasteurianum* DSM 525 at +0.045 V vs. SHE. In their study, they demonstrated a direct electron transfer mechanism between the microorganism and the cathode and a different metabolic pathway under electricity-driven fermentation to enhance butanol yield by up to 150%. Khosravanipour et al. [43] demonstrated the previous findings of Choi et al. [69] and studied the effect of the electrode materials, glucose concentration, temperature, and applied potential to optimize the butanol production by *Clostridium pasteurianum*. In their study, a maximum butanol concentration of 13.31 g.L^−1^ was achieved for an initial glucose concentration of 120 g.L^−1^ electro-fermented at 33.51 °C at a fixed potential of +1.32 V vs. Ag/AgCl.

1,3-propanediol (1,3-PDO) has raised considerable interest because of its extensive applications in the chemical industry (e.g., polymer synthesis, cosmetics, solvents, antifreeze, and lubricants) and expanding market [70]. Zhou et al. [71] studied the cathodic EF of glycerol to 1,3-PDO with a mixed population of fermenting glycerol microbes, demonstrating current enhances in 1,3-PDO production from 24.8% to 50.1% at −0.9 V vs. SHE as a result of the metabolic shift from propionate to 1,3-PDO.

## 5. Current Challenges and Prospects

As previously commented, EF shows an important potential to revalorize organic wastes of different natures into value-added products and biofuels. Despite the significant advances reported so far in lab-scale conditions, the transition to a pilot or industrial scale remains a distant horizon. The EF concept emerged ten years ago, so the number of electro-fermenters at the pilot scale is still reduced. One of the most important limitations is the size of the reactor which compromises the performance of the process and increases the energy requirements. It is crucial to maintain the efficiency of the electron transfer from the exoelectrogen to the anode considering that decreases as the anode size increases. This results in an increase in the cost of the hydrogen, or any other value-added compound produced [5,20,72,73]. In order to minimize energy losses, it is important to control the pH gradient which modifies the cathode potential as well as the exoelectrogen metabolism on the anodic surface. The ohmic losses also contribute to the energy consumption and can be reduced by increasing the conductivity of the membrane and the electrolyte as well as the stability of the electrodes [5,20]. As can be seen, the current limitations of scaling-up electro-fermenters are not so different from those exhibited in other BESs. However, the rapid evolution of the technology and its integration with other technologies such as MFCs, anaerobic digestion, or pyrolysis might help to address the current obstacles and favor its large-scale implementation.

## Figures and Tables

**Table 1:** Summary of the main advantages and disadvantages of fermentation and electro-fermentation.

	Fermentation	Electro-Fermentation
Advantages	Non-external energy consumption Developed technology	Good product control High yields for specific product Circular bioeconomy contribution Enhances microbial metabolism through electron transfer Potential higher productivity due to controlled electron flow
Disadvantages	Low product control Very much dependent on ambient conditions and microorganisms Low yields for specific products	Energy demanding Early-stage technology Higher cost (electrodes, catalysts, membrane, etc.) Needing precise control over microbial processes
Potential applications	Production of fermented food, alcoholic brewery, chemical products, etc.	Waste treatment and valorization Biochemical production Biofuel production Hydrogen production

**Table 2:** Summary of recent research work performed in single-chamber electro-fermenters.

Operation Mode	Electrode Materials	Applied Voltage (V)	Substrate	Biocatalyst	Main Products	Ref.
Batch	Cathode: graphite rod Anode: graphite brush	0.6	Glucose	Anaerobic sludge	CH_4_: 2998.4 mL	[35]
Semi-continuous	Ti/RuO_2_ mesh plate	0.4 to 1.0	Domestic wastewater	*Egeria densa*	CH_4_: 248.2 ± 21.0 mL.L^−1^.d^−1^	[36]
Batch	Graphite plate	−0.6 (vs. SCE)	Synthetic culture media	Anaerobic seed culture	CH_4_: 3.07 mmol	[37]
Fed-batch	Graphite	−0.6 (vs. Ag/AgCl)	Food waste	Sludge from sewage treatment plant	VFA: 4595 mg.L^−1^ H_2_ (26%) > CH_4_ (4%)	[38]
Fed-batch	Carbon cloth	±1.2 (vs. Ag/AgCl)	Glucose	*Thermotoga neapolitana*	H_2_: 9.91 mM AA: 0.75 g.L^−1^ LA: 0.35 g.L^−1^	[39]
Batch	Graphite	0.6 to 1.5	Glucose, acetate, and ethanol	Anaerobic sludge	BA: 0.38 g.L^−1^	[40]
Batch	Graphite	0.2, 0.4, 0.6 and 0.8	Pyruvate	*Bacillus subtilis*	SA: 0.83 g.L^−1^	[41]

VFA: Volatile Fatty Acids; AA: Acetic Acid; LA: Lactic Acid; BA: Butyric Acid; SA: Succinic Acid.

**Table 3:** Summary of recent research work performed in double-chamber electro-fermenters.

Operation Mode	Membrane	Electrode Materials	Applied Voltage (V)	Substrate	Biocatalyst	Main Products	Ref.
Semi-continuous	Nafion 117	Cathode: 8 carbon plates Anode: carbon bar	−0.8 V (vs. Ag/AgCl)	Thickened sewage sludge	Thickened sewage sludge	CH_4_: 3.57 L.L^−1^.day^−1^	[42]
Batch	Nafion 117	Gaphite felt	1.32 (−540 mV vs. Ag/AgCl)	Glucose	*C. pasteurianum*	Butanol: 13.31 g.L^−1^	[43]
Batch	Nafion 117	Graphite rod	−0.7 vs. SHE	Glucose, acetate, and ethanol	Anaerobic sludge from a mesophilic anaerobic digester	H_2_: 1.19 mol/mol of glucose BA: 0.3 g.L^−1^	[44]
Batch	Nafion 117	Anode: Platinized Ti Mesh Cathode: Carbon Cloth	−1 (vs. Ag/AgCl)	Lactate	Anaerobic sludge from dairy processing plant	PA: 0.9 g.L^−1^	[45]
Fed-batch	CMI7000	Graphite felt	−1, −0.6, −0.2 (vs. Ag/AgCl)	Glucose	Anaerobic sludge from wastewater treatment plant	CH_4_: 4.6 to 6.7 mL AA: 1.3 g.L^−1^ PA: 0.5 g.L^−1^ BA: 1.3 g.L^−1^	[46]

AA: Acetic Acid; BA: Butyric Acid; SA: Succinic Acid; PA: Propionic Acid.

## Data Availability

Not applicable.
